# MeiosisOnline: A Manually Curated Database for Tracking and Predicting Genes Associated With Meiosis

**DOI:** 10.3389/fcell.2021.673073

**Published:** 2021-08-13

**Authors:** Xiaohua Jiang, Daren Zhao, Asim Ali, Bo Xu, Wei Liu, Jie Wen, Huan Zhang, Qinghua Shi, Yuanwei Zhang

**Affiliations:** First Affiliated Hospital of USTC, Hefei National Laboratory for Physical Sciences at Microscale, School of Basic Medical Sciences, Division of Life Sciences and Medicine, CAS Center for Excellence in Molecular Cell Science, University of Science and Technology of China, Hefei, China

**Keywords:** meiosis, manual curation, data mining, Greed AUC Stepwise, database

## Abstract

Meiosis, an essential step in gametogenesis, is the key event in sexually reproducing organisms. Thousands of genes have been reported to be involved in meiosis. Therefore, a specialist database is much needed for scientists to know about the function of these genes quickly and to search for genes with potential roles in meiosis. Here, we developed “MeiosisOnline,” a publicly accessible, comprehensive database of known functional genes and potential candidates in meiosis (https://mcg.ustc.edu.cn/bsc/meiosis/index.html). A total of 2,052 meiotic genes were manually curated from literature resource and were classified into different categories. Annotation information was provided for both meiotic genes and predicted candidates, including basic information, function, protein–protein interaction (PPI), and expression data. On the other hand, 165 mouse genes were predicted as potential candidates in meiosis using the “Greed AUC Stepwise” algorithm. Thus, MeiosisOnline provides the most updated and detailed information of experimental verified and predicted genes in meiosis. Furthermore, the searching tools and friendly interface of MeiosisOnline will greatly help researchers in studying meiosis in an easy and efficient way.

## Background

Meiosis, the process to generate daughter cells with an intact, haploid genome through one round of DNA replication followed by two rounds of cell division, is a basic feature of sexual reproductive organisms (Gerton and Hawley, 2005; Miller et al., 2013; Bolcun-Filas and Handel, 2018; Biswas et al., 2021). Compared with mitosis, meiosis is characterized by homologous chromosome separation, which ensures the genetic integrity of all daughter cells (Sato et al., 2021). A series of biological processes would take place during meiosis prophase I to guarantee the formation and repair of programmed meiotic DNA double-strand breaks (DSBs) and the pair and synapsis between homologous chromosomes, as well as the formation of meiotic crossovers (Handel and Schimenti, 2010; Baudat et al., 2013; Gray and Cohen, 2016; Ranjha et al., 2018; Jiao et al., 2020; Li et al., 2021).

With the development of genomic technologies on model organisms and recent advances of transcriptomics and proteomics, tremendous articles have been published on meiosis from different species, and we get a clearer understanding about the genetic control of key events in meiosis (Watanabe et al., 2001; Wang et al., 2009; Chalmel and Rolland, 2015; Chen et al., 2018). However, information about meiotic genes is widely fragmented, which makes it still difficult to illuminate/highlight genes, molecular complexes, and/or signaling pathways involved in meiosis. What is more, it is still challenging to identify novel meiotic genes, especially in mammalian meiosis, since genetic modification in model organisms is time-consuming and is like a gamble sometimes (Khan et al., 2018, 2020; Huang et al., 2019; Xie et al., 2019; Yousaf et al., 2020). Thus, a specialist database that can provide integrated annotation of meiotic genes and predict novel functional genes is urgently needed.

Here, we report a publicly accessible, comprehensive database, MeiosisOnline.^1^ It is the first resource that is not only a well-structured repository of experimentally verified meiotic genes with detailed annotation, but also a powerful tool to predict genes that may function in meiosis.

## Materials and Methods

### Manual Curation of Literature

To collect the information of meiotic genes, specific keywords were used to search in PubMed (Supplementary Table 1). Then all the collected papers were curated manually and genes that had been validated by experiments were deemed as functional meiotic genes.

### Gene Expression Data Collection

Gene expression information was retrieved from the ArrayExpress database.^2^ Datasets from Affymetrix GeneChip platform were downloaded and were divided into different categories, including “developmental stages,” “gene disturbance,” “before and after treatment,” and “tissues and cell types” (Supplementary Table 2). Gene expression data combined with category information are provided as annotation information in MeiosisOnline and applied for predicting genes that may function in meiosis.

### Annotation

Annotation information for each gene in MeiosisOnline contains “basic information,” “function annotation and classification,” “protein–protein interaction (PPI) and gene expression.”

(1) Basic information: gene name/synonyms, nucleotide sequences, etc., were extracted from GenBank^3^ and UniProt Knowledgebase.^4^
(2) Function annotation and classification: detailed functional information is also manually collected from literature reports. (i) Which meiotic stage is the gene involved? (ii) Did the gene function in one sex or both sexes? (iii) Whether deletion or mutation of the gene in model organism has a phenotype in fertility? (iv) Which protein complex of the gene is involved? (v) The cellular location and expression pattern in tissues or cell lines. (vi) Experimental methods used for functional analysis. (vii) The information of related literature and figures for illustrating the function of protein/gene. (viii) Gene ontology annotation for collected genes.
(3) Protein–protein interaction and gene expression: both verified and predicted PPI information were provided. Gene expression pattern in reproductive system was also provided graphically.

### Implementation

To execute more jobs in parallel, a Dell 730 server with LAMP architecture is used to host the MeiosisOnline database. The server is equipped with 128 GB RAM and two 12-core Intel processors (2.2 GHz). The jQuery is used to render the interface and Python and R languages are employed to supply the backend.

## Results

### The Manual Curation of Meiosis-Related Genes From the Literature

MeiosisOnline is aiming to construct a functional annotation pipeline about meiosis-associated genes from published articles. After keywords querying in PubMed, about 45,000 research articles published before January 1, 2021, were collected. All collected papers were manually curated, and functional meiotic genes are only included as those with functional experimental validation (Supplementary Table 3). In total, 2,052 unique meiotic genes with experimentally verified functions from 84 species were curated along with functional information in MeiosisOnline. We found that the functional meiotic genes are firstly derived from mice, which accounts for 28.74% of the total reported genes, followed by human (5.16%) and rat (5.07%). Furthermore, other species comprise the rest of 61.03% (Supplementary Table 4). To be noted that the genes always have preferably expression profiles, for example, *Mlh3* (mg0000873) expresses during both male and female meiosis, *Sun5* (mg0000693) only presents in male germ cells, while *Bmp15* (mg0000982) is specially expressed in oocytes.

### The Overall Framework of MeiosisOnline

**FIGURE 1:**
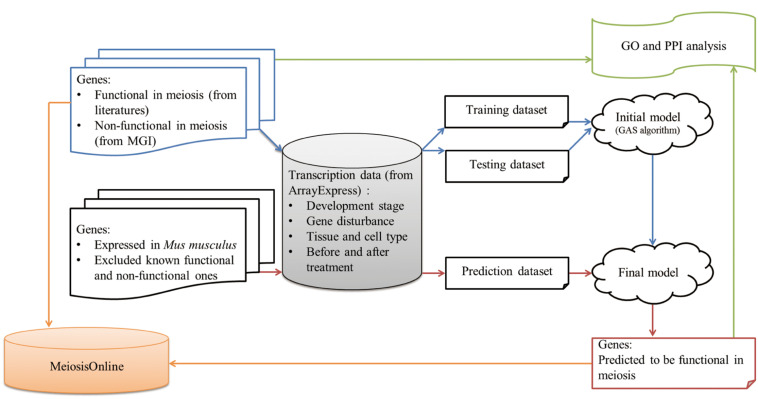
MeiosisOnline database scheme. Manually curated functional genes in meiosis are collected and further incorporated in the MeiosisOnline database.

MeiosisOnline is developed in a user-friendly manner and the major functional modules of the database (Figure 1) include:

#### Search Page

Users could find their interested genes using the Search page.^5^

Four additional search options were also provided^6^ :

(1) Advanced search. Users can query up to three keywords and set up different combination by selecting the operators (“and,” “or,” or “exclude”) to find the information more specifically (Supplementary Figure 1A).
(2) BLAST search. After protein sequence (FASTA format) uploading, it could map identical and homologous proteins recorded in MeiosisOnline database (Supplementary Figure 1B).
(3) Orthologous search. Simple orthologous search is specified in finding orthologs for designated genes, while advanced orthologous search is to display all orthologs between two selected species (Supplementary Figure 1C).
(4) Chromosome location. This module could list all genes in a given genome region (Supplementary Figure 1D).

#### Browse Page

In the Browse page, users can browse genes by classifications or species^7^ (Supplementary Figure 2A). Users can get all the genes belonging to a certain category in a tabular form. For example, users can browse MeiosisOnline Genes (MGs) collected from knockout mice (Supplementary Figure 2B) or MG genes identified in different species, e.g., *Homo sapiens* (Supplementary Figure 2C).

#### Candidates Page

In the Candidates page, MeiosisOnline lists all the predicted functional genes in mouse^8^ (Supplementary Figure 3A). Clicking the MG ID, detailed information for a candidate gene can be seen (Supplementary Figure 3B).

#### Feedback Page

Users can submit suggestions about the records integrated in MeiosisOnline or submit novel verified meiotic gene information to our database.^9^

### MeiosisOnline Integrates Information of Functional Genes in Meiosis

**FIGURE 2:**
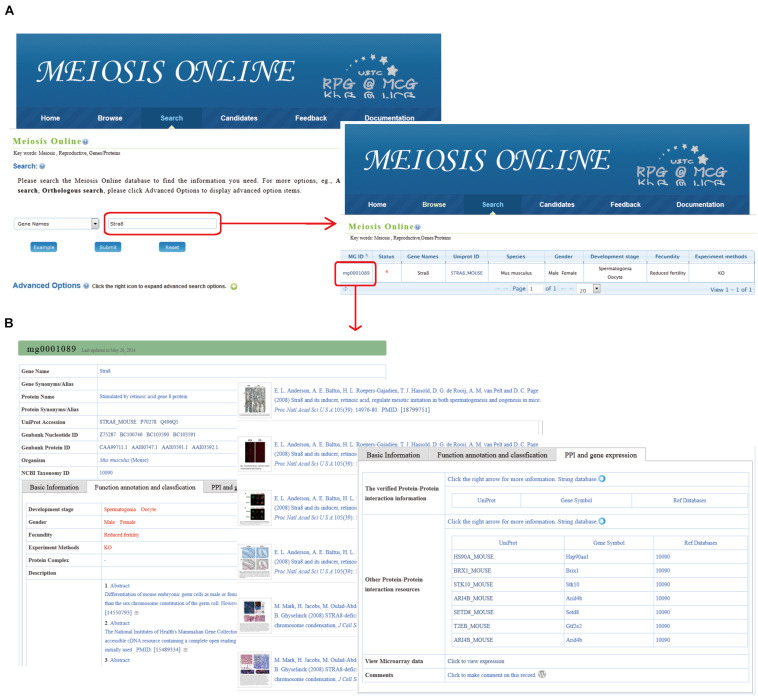
The search function of MeiosisOnline. **(A)** Users can simply input gene “*Stra8*” for querying. The results are shown in a tabular format. Users can visualize the detailed information by clicking on the MeiosisOnline ID (MG0001089). **(B)** The detailed information for mouse gene “*Stra8*.” The information presented has been manual checked and will be updated based on newly published data.

Besides the general information including gene ID, protein ID, taxonomy ID, and basic descriptions, MeiosisOnline also provides high-quality functional annotation for the collected genes. Based on the function annotation information, the experimental verified genes were classified into different categories. Additionally, figures and/or tables illustrating the function of the collected meiotic genes were also incorporated. Moreover, manually annotated functions, signaling pathways, and associated protein complexes of the collected meiotic genes were provided (Figure 2). The functional distribution of these genes in various stages of meiosis and fecundity is also listed (Supplementary Tables 4, 5).

For instance, using “*Stra8*” for query, results will be listed in tabular form, including MeiosisOnline Gene ID (MG ID), gene names, UniProt ID, etc. (Figure 2A). Once clicking the MG ID (MG0001089), detailed information for mouse *Stra8* is available that includes the following: (1) basic information (gene name, nucleotide and protein sequences, etc.), (2) functional annotation and classification from related literature (developmental stages, experimental methods, literature abstract, relevant figures, etc.), and (3) PPI and gene expression information (Figure 2B).

### MeiosisOnline Facilitates the Discovery of Functional Meiotic Genes

To expand the utilization of our MeiosisOnline database, a prediction model was constructed and used to predict the candidate functional meiotic genes. As mouse is one of the best studied animal models, the GAS algorithm (Zhang et al., 2013) was used to predict potential meiotic functional genes from *Mus musculus* (Supplementary Figure 4).

To verify the efficiency of GAS, we randomly separated the training data into two equal parts: one as a new training dataset and the other as a testing dataset. The model was split into three stages: stage 1 was constructed with features of the category “developmental stages,” stage 2 included features from the categories “tissues and cell types” and “before and after treatment,” and stage 3 is the features of the category “gene disturbance.” As shown in Supplementary Figure 5, the performances of GAS models were better when more features were added to them. Then, based on the experimentally verified meiotic genes and gene expression data, 590 mouse genes with experimentally verified function in meiosis were used as the positive training dataset. The negative training dataset contained 5,868 genes from MGI (Mouse Genome Informatics),^10^ of which knockout mice did not have any abnormalities in the reproductive system. Three hundred and ninety-four features used for GAS construction and prediction were extracted from the 85 microarray data (Supplementary Table 2).

Ultimately, 165 candidate genes (GAS probability > 0.5) having potential role were sorted out and annotated in MeiosisOnline (see text footnote 8). For the candidate genes, information that implicate their function in meiosis, including gene expression, protein localization, structure, and protein interactions, are included in MeiosisOnline.

**FIGURE 3:**
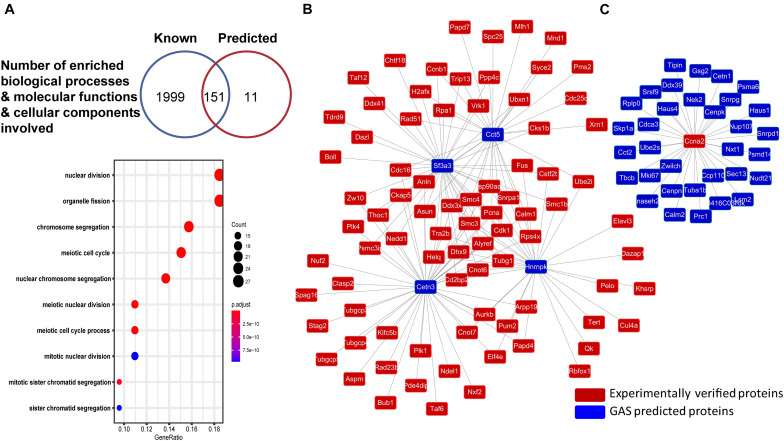
The GO analysis and protein network in MeiosisOnline. **(A)** GO analysis for known and predicted genes in MeiosisOnline and GO enrichment analysis of the predicted genes. **(B,C)** The examples of potential protein network of meiosis analyzed by MeiosisOnline.

We also performed GO enrichment analysis on both literature-reported genes in *M. musculus* (Supplementary Table 6) and GAS-predicted candidate genes (Supplementary Table 7). Compared with whole genome data, we statistically calculated the distribution of MGs in cellular components, biological processes, and molecular functions by R (hypergeometric distribution, *p* < 0.05) (Yu et al., 2012). Among all GO terms, 118 in biological processes, 1 in molecular functions, and 32 in cellular components are overlapped in both sets of reported and predicted genes (Figure 3A). Considering that predicted genes enriched by overlapped GO term have more potential in regulating meiosis, interestingly, meiotic cell cycle (GO: 0051321) was enriched from both sets of genes (reported and predicted) (Supplementary Table 8).

Furthermore, as genes are mostly regulated through network structure in meiosis, we mapped out the PPIs among all of the genes and constructed a potential meiosis network with 1,083,566 reported PPIs among 26,569 proteins in MeiosisOnline. For example, *Cct5* and *Sf3a3*, which have not been reported, show very high connectivity with reported genes (Figure 3B). Further investigation of *Cct5* and *Sf3a3* would disclose how these two genes interacted with reported genes and what is the function of these interactions. Moreover, we also found that reported genes like *Ccna2* interacted wildly with predicted genes (Figure 3C).

## Discussion

Studies on animal models, especially genetically modified mice, have revealed many critical regulators involved in meiosis (Marston and Amon, 2004; Handel and Schimenti, 2010; Robert et al., 2016; Jiang et al., 2017, 2018); however, the information for these meiotic genes are scattered among thousands of papers. Thus, it is difficult to collect and compare the information of meiotic genes among different species from papers. Here, based on manual curation of meiosis-related genes from the literature, the first comprehensive database, MeiosisOnline, focusing on meiosis was developed.

As the fundamental process of gametogenesis, systematical annotation for meiotic genes is important to conduct further experiment study. Currently, only a few databases provided information related to meiosis, with limited features. Some databases were only repositories of gene expression data such as GermOnline 4.0 (Lardenois et al., 2010), SpPress (Vibranovski et al., 2009), and GermSAGE (Lee et al., 2009), and the utilization of these databases to obtain valuable information regarding experimentally verified function is not satisfactory. Some of those are limited to a specific species or a certain biological process, such as SpPress that focused only on spermatogenesis in *Drosophila* (Vibranovski et al., 2009), ReCGiP that focused on reproduction in pig (Yang et al., 2010), and MeioBase that focused on meiotic genes in plant (Li et al., 2014). In our study, MeiosisOnline provided detailed and comprehensive information and annotation of the meiotic genes, including basic information, functional annotation and classification, PPI, and gene expression, etc. With these diverse information, users can easily access the detailed functional information of meiosis-associated genes.

Additionally, besides the proven functional meiotic genes, among the 2,300 genes that are predominantly expressed in the testis (Schultz et al., 2003), the function of most genes in meiosis are still unclear. In MeiosisOnline, based on literature-reported genes and genome-wide transcriptional data from ArrayExpress analysis, 165 genes (GAS probability > 0.5) in mouse are predicted to be involved in meiosis. As we know that homologous recombination is the basic feature of meiosis (Zickler and Kleckner, 2015), when we perform GO terms for the predicted genes by MeiosisOnline, double-strand break repair *via* homologous recombination (GO: 0000724) was one of the most enriched GO (Supplementary Table 8), which implies that these predicted genes may function during meiosis and further functional study by animal models should be conducted.

What is more, MeiosisOnline could conduct the study focus on complex molecular and/or signaling networks in meiosis. During meiosis, some genes are regulated through the network structure; for example, the deletion of *Hadc1* or *Ddac2* alone did not affect meiosis, while their combined deletion resulted in meiotic arrest and subsequent oocyte depletion (Ma et al., 2012). Hence, mapping out PPIs among known and predicted genes is useful in uncovering the novel regulating mechanism of meiosis.

In summary, MeiosisOnline is the first specialist database on meiosis. It not only provides comprehensive information for experimental verified meiotic genes but can also predict genes that may function in meiosis. It would be a helpful resource for researchers to gain a new insight in meiosis.

## Data Availability Statement

The original contributions presented in the study are included in the article/Supplementary Material, further inquiries can be directed to the corresponding author/s.

## Author Contributions

YZ and QS conceived and supervised the project. XJ, HZ, AA, and WL collected the data from the literature and ArrayExpress, as well as positive and negative training data for the prediction of potential meiotic functional genes. DZ developed the web interface. XJ, DZ, YZ, and QS wrote the manuscript. JW reviewed the manuscript. All authors contributed to the article and approved the submitted version.

## Conflict of Interest

The authors declare that the research was conducted in the absence of any commercial or financial relationships that could be construed as a potential conflict of interest.

## Publisher’s Note

All claims expressed in this article are solely those of the authors and do not necessarily represent those of their affiliated organizations, or those of the publisher, the editors and the reviewers. Any product that may be evaluated in this article, or claim that may be made by its manufacturer, is not guaranteed or endorsed by the publisher.

## Footnotes

1
https://mcg.ustc.edu.cn/bsc/meiosis/index.html

2
http://www.ebi.ac.uk/arrayexpress/

3
http://www.ncbi.nlm.nih.gov/genbank

4
http://www.uniprot.org/

5
http://mcg.ustc.edu.cn/bsc/meiosis/search.html

6
http://mcg.ustc.edu.cn/bsc/meiosis/advanced_search.html

7
https://mcg.ustc.edu.cn/bsc/meiosis/browse.html

8
http://mcg.ustc.edu.cn/bsc/meiosis/prediction.php

9
http://mcg.ustc.edu.cn/bsc/meiosis/feedback.html

10
http://www.informatics.jax.org/

## Supplementary Material

Supplementary Figure 1 The advanced options of MeiosisOnline. **(A)** Advanced search which allows users to simultaneously input three terms for querying; **(B)** BLAST search used for protein sequence querying; **(C)** orthologous search. Browse orthologs for a gene in all species by simple search or browse orthologs of all genes in two species by advanced search. An example of pairwise orthologous browsing in human and mice were showed; **(D)** searching the MG genes based on chromosome and/or chromosomal location.

Supplementary Figure 2 **(A)** All entries of MeiosisOnline can be browsed through taxonomic hierarchy and manual classifications; **(B)** by MG genes collected from KO mouse model, and **(C)** by MG genes identified in *Homo sapiens*.

Supplementary Figure 3 **(A)** The candidate genes that are predicted by GAS model; **(B)** the detailed information of the predicted example gene “mg0002027.”

Supplementary Figure 4 The pseudo code illustrating the GAS algorithm.

Supplementary Figure 5 The performance of GAS regarding its sensitivity (*Sn*), specificity (*Sp*), accuracy (*Ac*), Matthews correlation coefficient (MCC), and area under cover (AUC). Increase in the number of selected features resulted in improved AUC value.

## References

[B1] Baudat F. Imai Y. De Massy B. (2013). Meiotic recombination in mammals: localization and regulation. *Nat. Rev. Genet.* 14 794–806. 10.1038/nrg3573 24136506

[B2] Biswas L. Tyc K. El Yakoubi W. Morgan K. Xing J. Schindler K. (2021). Meiosis interrupted: the genetics of female infertility via meiotic failure. *Reproduction* 161 R13–R35. 33170803 10.1530/REP-20-0422 PMC7855740

[B3] Bolcun-Filas E. Handel M. A. (2018). Meiosis: the chromosomal foundation of reproduction. *Biol. Reprod.* 99 112–126. 10.1093/biolre/ioy021 29385397

[B4] Chalmel F. Rolland A. D. (2015). Linking transcriptomics and proteomics in spermatogenesis. *Reproduction* 150 R149–R157. 26416010 10.1530/REP-15-0073

[B5] Chen Y. Zheng Y. Gao Y. Lin Z. Yang S. Wang T. (2018). Single-cell RNA-seq uncovers dynamic processes and critical regulators in mouse spermatogenesis. *Cell Res.* 28 879–896. 10.1038/s41422-018-0074-y 30061742 PMC6123400

[B6] Gerton J. L. Hawley R. S. (2005). Homologous chromosome interactions in meiosis: diversity amidst conservation. *Nat. Rev. Genet.* 6 477–487. 10.1038/nrg1614 15931171

[B7] Gray S. Cohen P. E. (2016). Control of meiotic crossovers: from double-strand break formation to designation. *Annu. Rev. Genet.* 50 175–210. 10.1146/annurev-genet-120215-035111 27648641 PMC5319444

[B8] Handel M. A. Schimenti J. C. (2010). Genetics of mammalian meiosis: regulation, dynamics and impact on fertility. *Nat. Rev. Genet.* 11 124–136. 10.1038/nrg2723 20051984

[B9] Huang Z. Khan M. Xu J. Khan T. Ma H. Khan R. (2019). The deubiquitinating gene Usp29 is dispensable for fertility in male mice. *Sci. China Life Sci.* 62 544–552. 10.1007/s11427-018-9469-4 30919279

[B10] Jiang H. Gao Q. Zheng W. Yin S. Wang L. Zhong L. (2018). MOF influences meiotic expansion of H2AX phosphorylation and spermatogenesis in mice. *PLoS Genet.* 14:e1007300. 10.1371/journal.pgen.1007300 29795555 PMC6019819

[B11] Jiang L. Li T. Zhang X. Zhang B. Yu C. Li Y. (2017). rpl10l is required for male meiotic division by compensating for RPL10 during meiotic sex chromosome inactivation in mice. *Curr. Biol.* 27 1498–1505.e1496. 28502657 10.1016/j.cub.2017.04.017

[B12] Jiao Y. Fan S. Jabeen N. Zhang H. Khan R. Murtaza G. (2020). A TOP6BL mutation abolishes meiotic DNA double-strand break formation and causes human infertility. *Sci. Bull* 65 2120–2129. 10.1016/j.scib.2020.08.026 36732965

[B13] Khan M. Jabeen N. Khan T. Hussain H. M. J. Ali A. Khan R. (2018). The evolutionarily conserved genes: Tex37, Ccdc73, Prss55 and Nxt2 are dispensable for fertility in mice. *Sci. Rep.* 8:4975. 10.1038/s41598-018-23176-x PMC5862965 29563520

[B14] Khan R. Ye J. Yousaf A. Shah W. Aftab A. Shah B. (2020). Evolutionarily conserved and testis-specific gene, 4930524B15Rik, is not essential for mouse spermatogenesis and fertility. *Mol. Biol. Rep.* 47 5207–5213. 10.1007/s11033-020-05595-0 32592116

[B15] Lardenois A. Gattiker A. Collin O. Chalmel F. Primig M. (2010). GermOnline 4.0 is a genomics gateway for germline development, meiosis and the mitotic cell cycle. *Database* 2010:baq030. 10.1093/database/baq030 21149299 PMC3004465

[B16] Lee T. L. Cheung H. H. Claus J. Sastry C. Singh S. Vu L. (2009). GermSAGE: a comprehensive SAGE database for transcript discovery on male germ cell development. *Nucleic Acids Res.* 37 D891–D897. 18832368 10.1093/nar/gkn644 PMC2686471

[B17] Li H. Meng F. Guo C. Wang Y. Xie X. Zhu T. (2014). MeioBase: a comprehensive database for meiosis. *Front. Plant Sci.* 5:728. 10.3389/fpls.2014.00728 25566299 PMC4267189

[B18] Li Y. Wu Y. F. Jiang H. W. Khan R. Han Q. Q. Iqbal F. (2021). The molecular control of meiotic double-strand break (DSB) formation and its significance in human infertility. *Asian J. Androl.* 10.4103/aja.aja_5_21 PMC8577252 33586697

[B19] Ma P. Pan H. Montgomery R. L. Olson E. N. Schultz R. M. (2012). Compensatory functions of histone deacetylase 1 (HDAC1) and HDAC2 regulate transcription and apoptosis during mouse oocyte development. *Proc. Natl. Acad. Sci. U.S.A.* 109 E481–E489. 22223663 10.1073/pnas.1118403109 PMC3286984

[B20] Marston A. L. Amon A. (2004). Meiosis: cell-cycle controls shuffle and deal. *Nat. Rev. Mol. Cell Biol.* 5 983–997. 10.1038/nrm1526 15573136

[B21] Miller M. P. Amon A. Unal E. (2013). Meiosis I: when chromosomes undergo extreme makeover. *Curr. Opin. Cell Biol.* 25 687–696. 10.1016/j.ceb.2013.07.009 23916768 PMC3836829

[B22] Ranjha L. Howard S. M. Cejka P. (2018). Main steps in DNA double-strand break repair: an introduction to homologous recombination and related processes. *Chromosoma* 127 187–214. 10.1007/s00412-017-0658-1 29327130

[B23] Robert T. Nore A. Brun C. Maffre C. Crimi B. Bourbon H. M. (2016). The TopoVIB-Like protein family is required for meiotic DNA double-strand break formation. *Science* 351 943–949. 10.1126/science.aad5309 26917764

[B24] Sato M. Kakui Y. Toya M. (2021). Tell the difference between mitosis and meiosis: interplay between chromosomes, cytoskeleton, and cell cycle regulation. *Front. Cell Dev. Biol.* 9:660322. 10.3389/fcell.2021.660322 33898463 PMC8060462

[B25] Schultz N. Hamra F. K. Garbers D. L. (2003). A multitude of genes expressed solely in meiotic or postmeiotic spermatogenic cells offers a myriad of contraceptive targets. *Proc. Natl. Acad. Sci. U.S.A.* 100 12201–12206. 10.1073/pnas.1635054100 14526100 PMC218736

[B26] Vibranovski M. D. Lopes H. F. Karr T. L. Long M. (2009). Stage-specific expression profiling of Drosophila spermatogenesis suggests that meiotic sex chromosome inactivation drives genomic relocation of testis-expressed genes. *PLoS Genet.* 5:e1000731. 10.1371/journal.pgen.1000731 19936020 PMC2770318

[B27] Wang Z. Gerstein M. Snyder M. (2009). RNA-Seq: a revolutionary tool for transcriptomics. *Nat. Rev. Genet.* 10 57–63. 10.1038/nrg2484 19015660 PMC2949280

[B28] Watanabe Y. Yokobayashi S. Yamamoto M. Nurse P. (2001). Pre-meiotic S phase is linked to reductional chromosome segregation and recombination. *Nature* 409 359–363. 10.1038/35053103 11201746

[B29] Xie Y. Khan R. Wahab F. Hussain H. M. J. Ali A. Ma H. (2019). The testis-specifically expressed Dpep3 is not essential for male fertility in mice. *Gene* 711:143925. 10.1016/j.gene.2019.06.015 31212048

[B30] Yang L. Zhang X. Chen J. Wang Q. Wang L. Jiang Y. (2010). ReCGiP, a database of reproduction candidate genes in pigs based on bibliomics. *Reprod. Biol. Endocrinol.* 8:96. 10.1186/1477-7827-8-96 20707928 PMC3224910

[B31] Yousaf A. Wu Y. Khan R. Shah W. Khan I. Shi Q. (2020). Normal spermatogenesis and fertility in Ddi1 (DNA damage inducible 1) mutant mice. *Reprod. Biol.* 20 520–524. 10.1016/j.repbio.2020.08.006 33092996

[B32] Yu G. Wang L. G. Han Y. He Q. Y. (2012). clusterProfiler: an R package for comparing biological themes among gene clusters. *OMICS* 16 284–287. 10.1089/omi.2011.0118 22455463 PMC3339379

[B33] Zhang Y. Zhong L. Xu B. Yang Y. Ban R. Zhu J. (2013). SpermatogenesisOnline 1.0: a resource for spermatogenesis based on manual literature curation and genome-wide data mining. *Nucleic Acids Res.* 41 D1055–D1062. 23193286 10.1093/nar/gks1186 PMC3531227

[B34] Zickler D. Kleckner N. (2015). Recombination, pairing, and synapsis of homologs during meiosis. *Cold Spring Harb. Perspect. Biol.* 7:a016626. 10.1101/cshperspect.a016626 25986558 PMC4448610

