# Supplementary figures and images for: MeiosisOnline: A Manually Curated Database for Tracking and Predicting Genes Associated With Meiosis
Source: Front Cell Dev Biol. 2021 Aug 13;9:673073. doi: 10.3389/fcell.2021.673073 (PMC8415030; doi:10.3389/fcell.2021.673073)

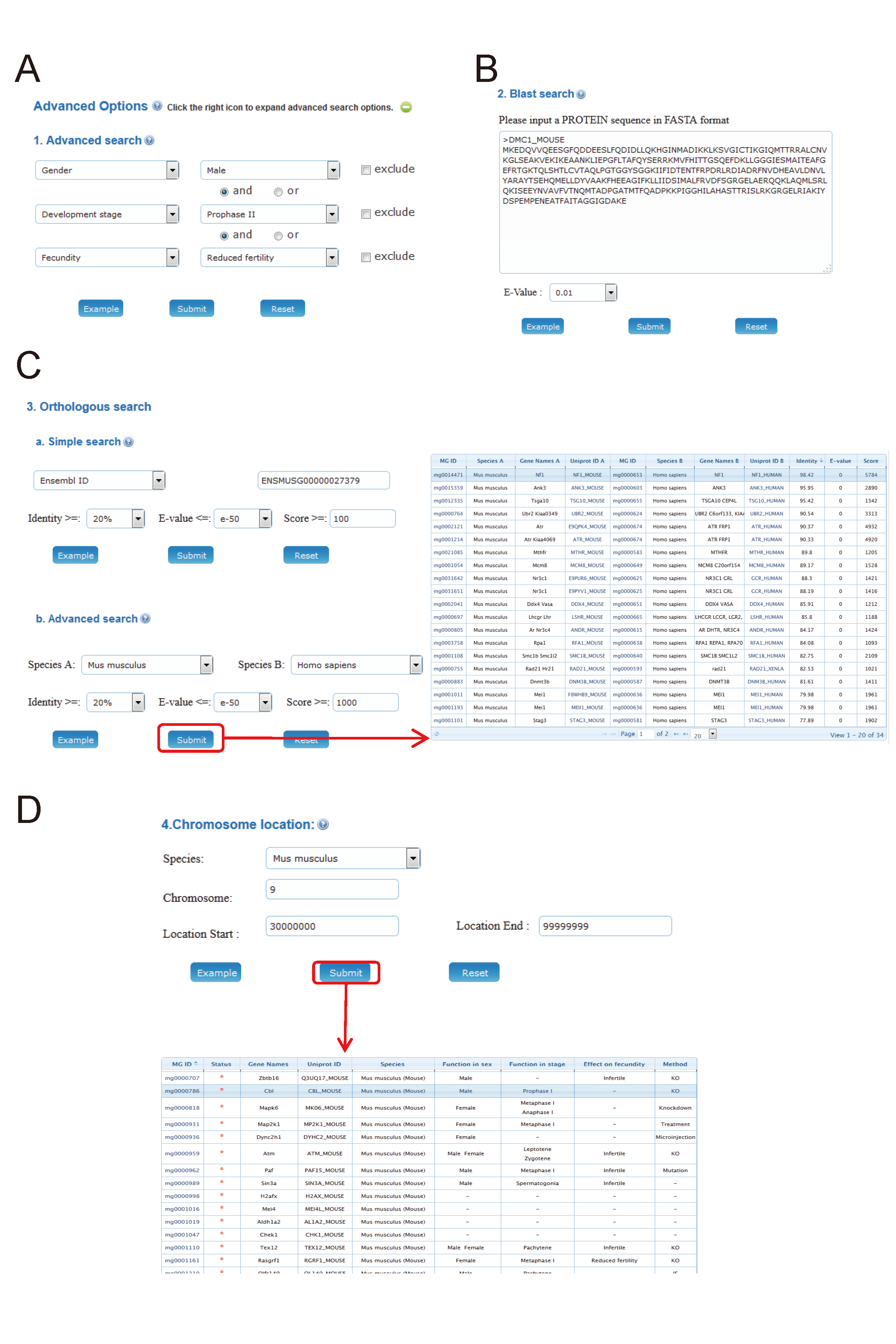

Supplement: Supplementary Figure 1 — The advanced options of MeiosisOnline. (A) Advanced search which allows users to simultaneously input three terms for querying; (B) BLAST search used for protein sequence querying; (C) orthologous search. Browse orthologs for a gene in all species by simple search or browse orthologs of all genes in two species by advanced search. An example of pairwise orthologous browsing in human and mice were showed; (D) searching the MG genes based on chromosome and/or chromosomal location. [file Image_1.TIF]

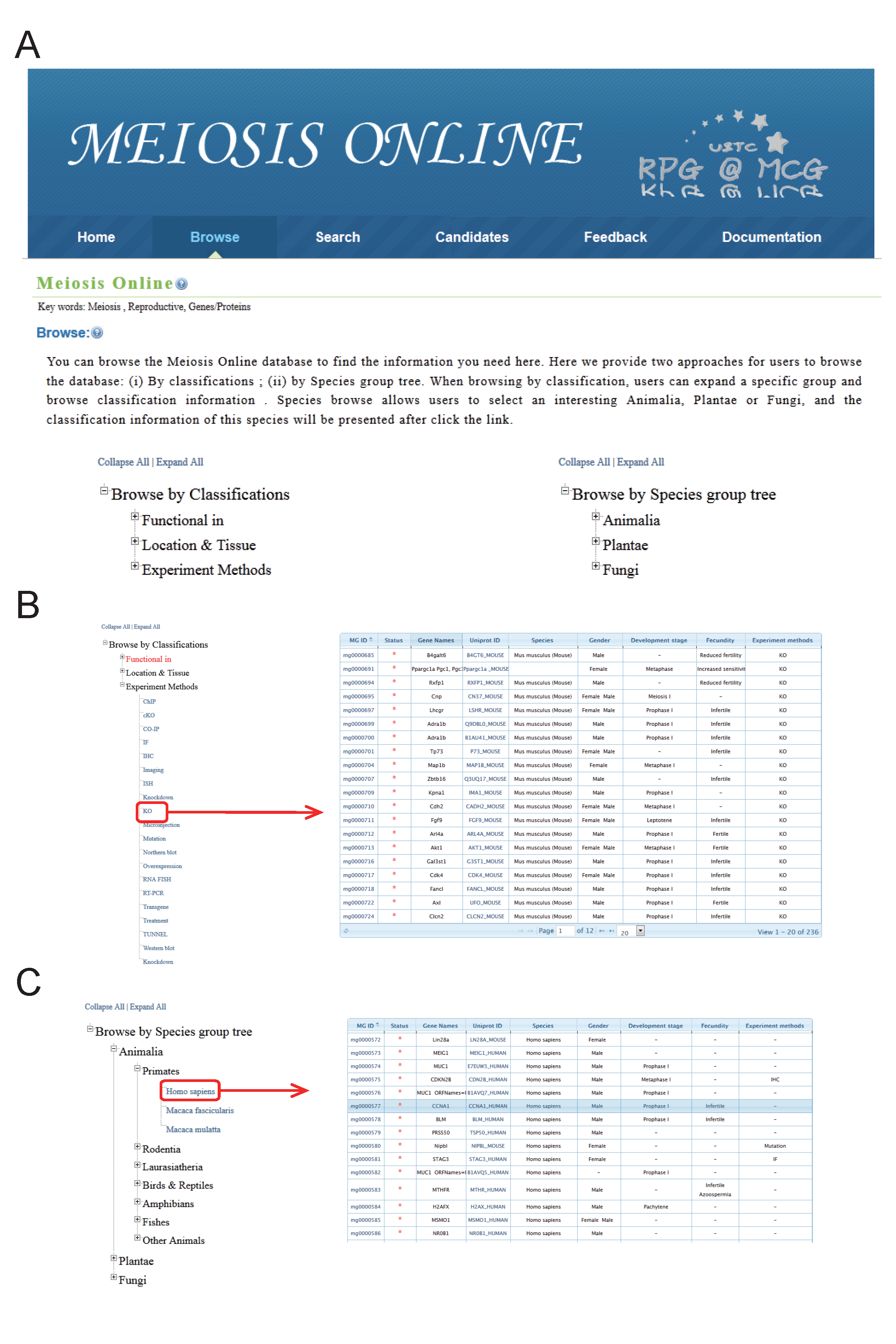

Supplement: Supplementary Figure 2 — (A) All entries of MeiosisOnline can be browsed through taxonomic hierarchy and manual classifications; (B) by MG genes collected from KO mouse model, and (C) by MG genes identified in Homo sapiens. [file Image_2.TIFF]

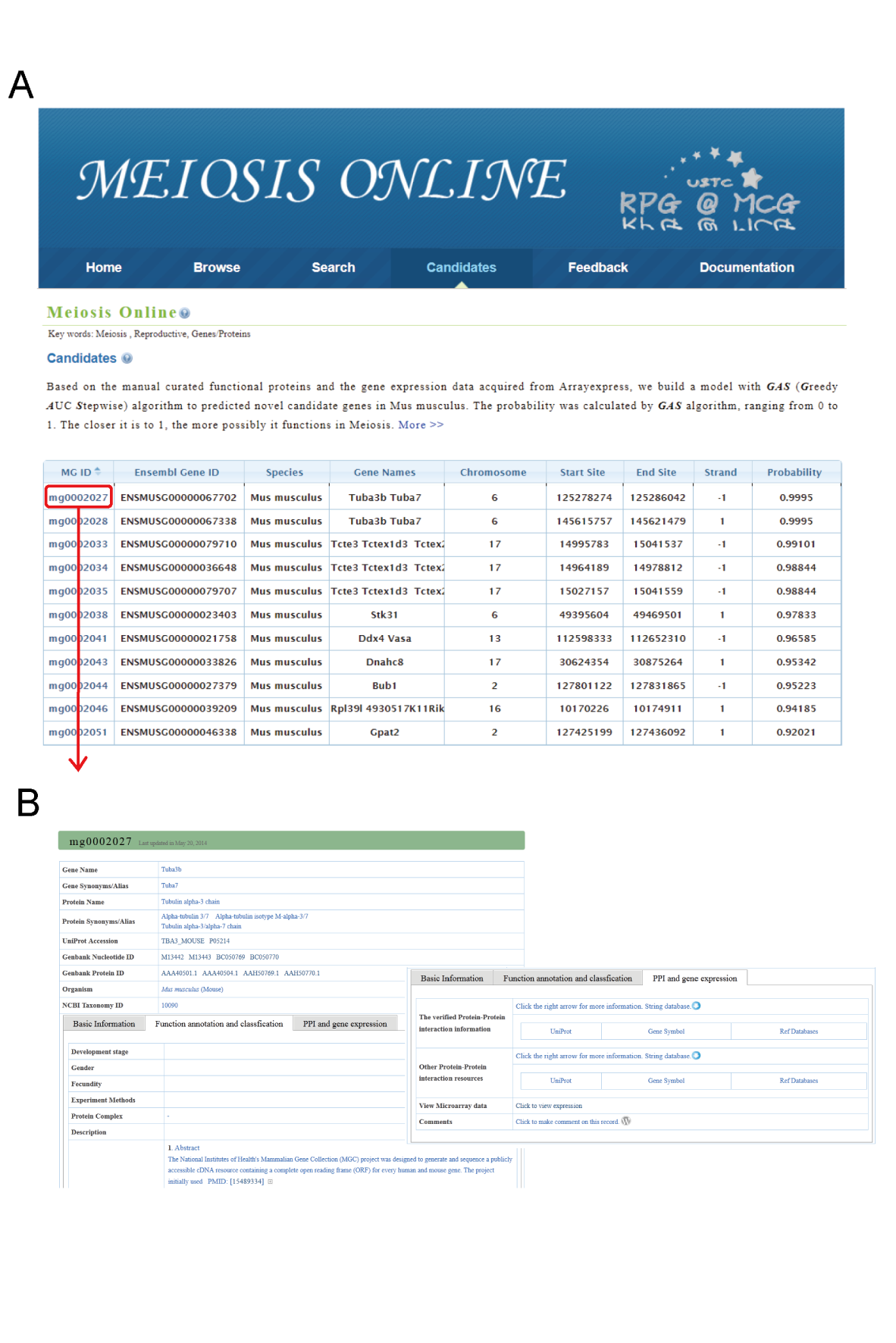

Supplement: Supplementary Figure 3 — (A) The candidate genes that are predicted by GAS model; (B) the detailed information of the predicted example gene “mg0002027.” [file Image_3.tif]

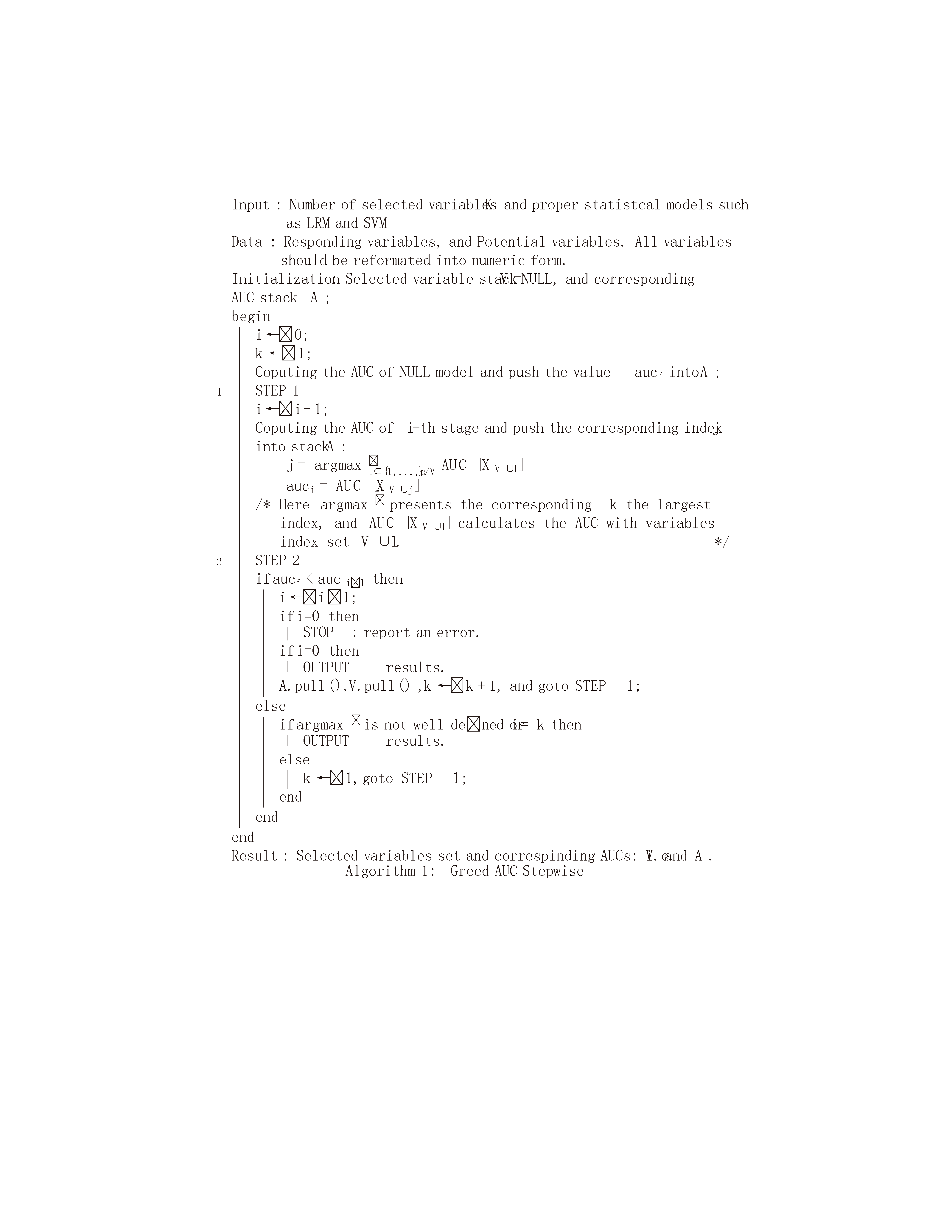

Supplement: Supplementary Figure 4 — The pseudo code illustrating the GAS algorithm. [file Image_4.TIF]

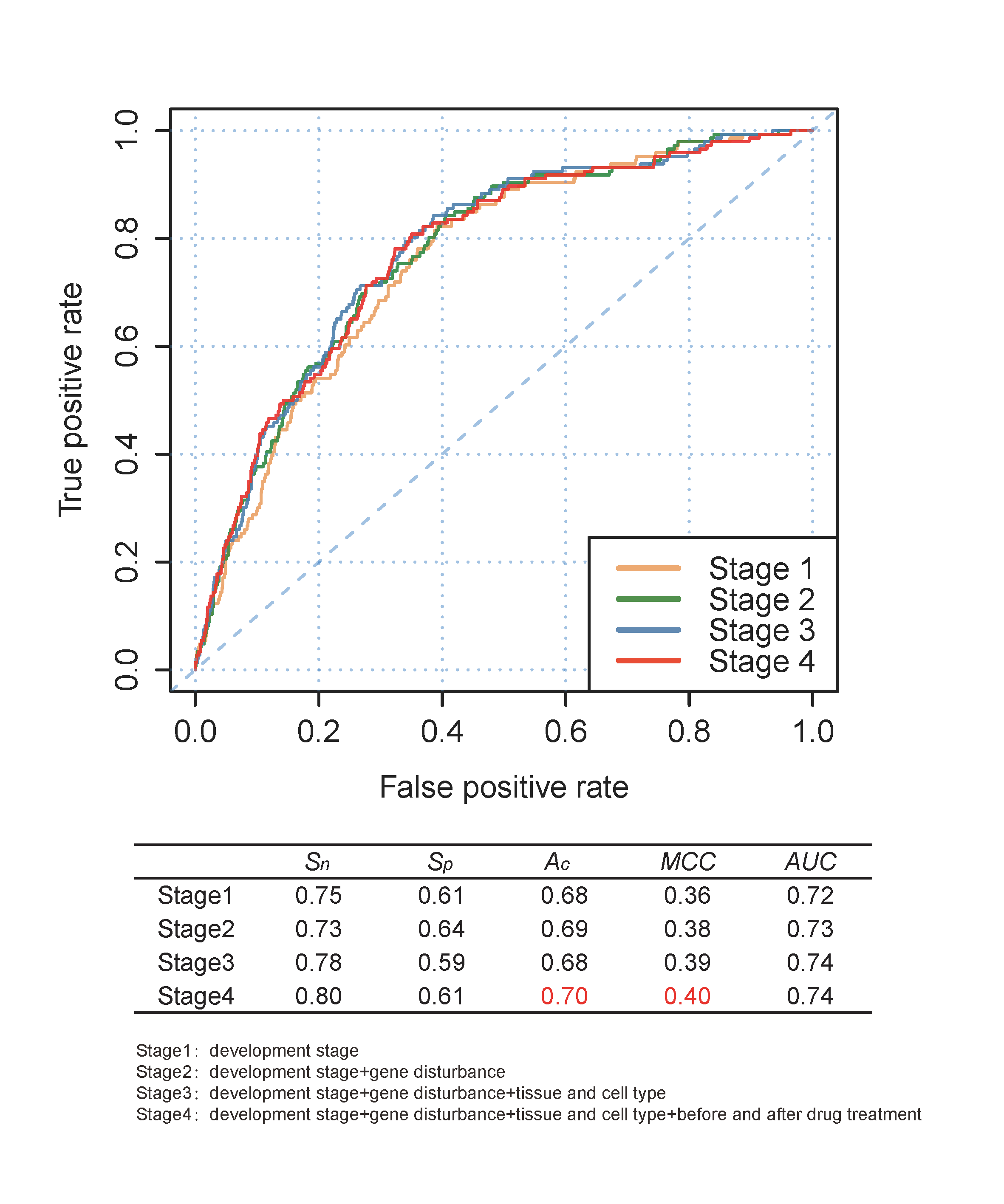

Supplement: Supplementary Figure 5 — The performance of GAS regarding its sensitivity (Sn), specificity (Sp), accuracy (Ac), Matthews correlation coefficient (MCC), and area under cover (AUC). Increase in the number of selected features resulted in improved AUC value. [file Image_5.TIFF]
